# A continuum-mechanical skeletal muscle model including actin-titin interaction predicts stable contractions on the descending limb of the force-length relation

**DOI:** 10.1371/journal.pcbi.1005773

**Published:** 2017-10-02

**Authors:** Thomas Heidlauf, Thomas Klotz, Christian Rode, Tobias Siebert, Oliver Röhrle

**Affiliations:** 1 Institute of Applied Mechanics (CE), University of Stuttgart, Stuttgart, Germany; 2 Stuttgart Research Centre for Simulation Technology (SRC SimTech), University of Stuttgart, Stuttgart, Germany; 3 Institute of Motion Science, Friedrich-Schiller-University, Jena, Germany; 4 Department of Sport and Motion Science, University of Stuttgart, Stuttgart, Germany; University of Virginia, UNITED STATES

## Abstract

Contractions on the descending limb of the total (active + passive) muscle force—length relationship (i. e. when muscle stiffness is negative) are expected to lead to vast half-sarcomere—length inhomogeneities. This is however not observed in experiments—vast half-sarcomere—length inhomogeneities can be absent in myofibrils contracting in this range, and initial inhomogeneities can even decrease. Here we show that the absence of half-sarcomere—length inhomogeneities can be predicted when considering interactions of the semi-active protein titin with the actin filaments. Including a model of actin—titin interactions within a multi-scale continuum-mechanical model, we demonstrate that stability, accurate forces and nearly homogeneous half-sarcomere lengths can be obtained on the descending limb of the static total force—length relation. This could be a key to durable functioning of the muscle because large local stretches, that might harm, for example, the transverse-tubule system, are avoided.

## Author summary

Muscle force generation is a complex process depending on muscle length, activation, and other time-dependent properties. Contractions on the descending limb of the force—length relation (i. e. when the maximum isometric force decreases with increasing muscle length) are interesting, since this behaviour is expected to result in non-physiological length inhomogeneities of the muscle microstructure. Previously, different mechanisms have been suggested that could have a stabilising effect on the muscle microstructure. However, superposition of several phenomena makes it difficult to separate the influence of a single mechanism in an experimental setup. Within a model, individual phenomena can be knocked out to quantify the influence of a single process. Here we show, using a physiologically motivated model of the whole muscle, that variable mechanical properties of the large protein titin (controlled by calcium as a second messenger) can guarantee stability of the muscle microstructure during contractions on the descending limb of the force—length relation.

## Introduction

The isometric active force—length relation of muscle fibres [1] and many muscles [2–4] is composed of strikingly linear segments. Based on this observation, the sarcomere microstructure, and the sliding filament theory [5, 6], a geometric model of the filament overlap has been established and validated [1], which shaped our understanding of muscle structure and functioning.

**Fig 1:**
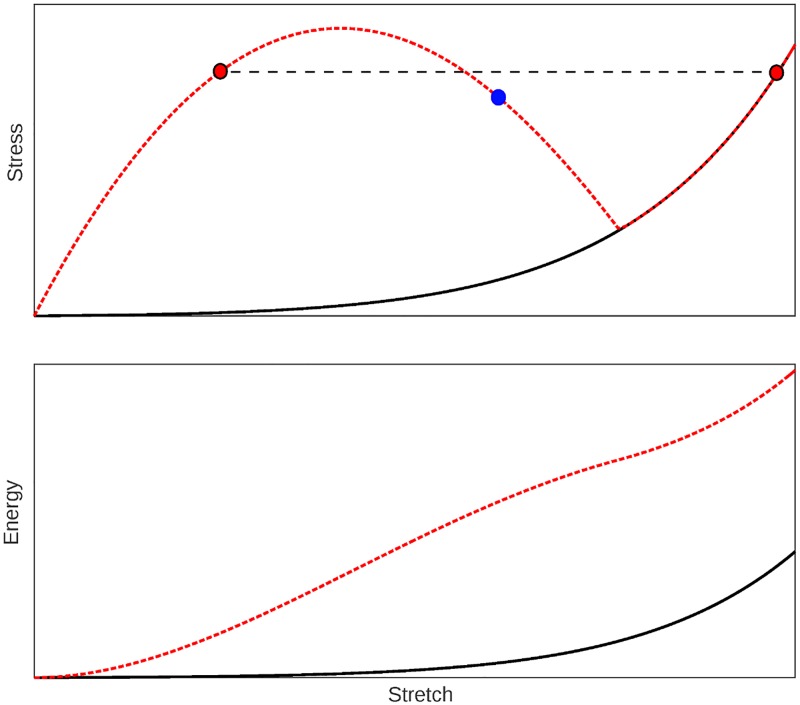
Schematic of passive and fully activated stress–stretch relations and corresponding energy—stretch relations. Top: Consider the case that muscle force is completely described by the passive stress (black solid curve) and total isometric stress (red dashed curve). Then, a fixed-length contraction of two in-series-arranged half-sarcomeres on the descending limb of the total stress—stretch relation (blue dot) is unstable. Small initial half-sarcomere length differences lead to a stronger, shorter half-sarcomere and a weaker, longer half-sarcomere. Hence, the short half-sarcomere will shorten further, stretching the longer half-sarcomere until static force equilibrium is established again on ascending albeit different limbs of the total stress—stretch relation (red dots). Such behaviour is not observed in experiments [8, 9]. Bottom: Passive, convex energy (black curve) and total, non-convex energy (red dashed curve).

From a mechanical point of view, the negative slope of the force—length relationship at long muscle lengths (descending limb) was and is of special interest for muscle physiologists and modellers due to its inherent instability. It has been suggested early [7] that contractions on the descending limb of the force—length relation will result in unstable behaviour leading to the formation of short and long half-sarcomeres in series during fixed-length muscle contractions (cf. Fig 1 top).

A number of human and animal skeletal muscles work on the descending limb of the active force—length relation [10]. Moreover, passive forces in single muscle fibres [11–13] and some entire skeletal muscles, e. g. the rabbit extensor digitorum longus, extensor digitorum II, and soleus [3, 4] or the frog semitendinosus [14], appear only at lengths that correspond to the descending limb of the active force—length relation [13, 15] leading to a descending limb in the total force—length relation. Simulation of these muscles is particularly challenging using continuum-mechanical finite element models, which typically superimpose the passive stress tensor with an active stress that includes the active force—length relation [16, 17]. The descending limb of the resulting total stress—stretch relation (Fig 1 top) causes instability of these models, which explains why previous continuum-mechanical models focused on muscles with significant passive forces occurring at short muscle length, i. e., that have no descending limb in the total force—length relation [17–21].

With respect to the above described approach of superimposing passive and active stress contributions, two aspects require further consideration. First, the energy corresponding to a total stress—stretch relation with a descending limb is described by a non-convex function, cf. Fig 1 (bottom), whereas a unique solution requires convexity. Second, the force—length relation is a static muscle property and, in light of muscle contraction phenomena like force enhancement (muscle force is increased after stretch compared to the corresponding isometric muscle force at the same final length) [22–24], it should not be used as a dynamic one [25]. Thus, the force—length relation should not be interpreted as a hyperelastic stress—stretch relation.

Unstable half-sarcomere behaviour on the descending limb can be mitigated by the damping effect of the force—velocity relation [26] and might possibly even be prevented by the interaction of the semi-active protein titin with the actin myofilament [27–29]. By calcium-induced actin—titin binding within the isotropic band of the half-sarcomere, the molecular spring length of titin would dramatically reduce. This would lead to an increased passive force when the (weaker) half-sarcomere is lengthened, which may prevent the evolution of a heterogeneous half-sarcomere—length distribution and instability on the descending limb of the force—length relation.

Recently (see [30]), we included a biophysical model describing force enhancement based on actin—titin interaction [31] in a continuum-mechanical finite element model [19, 32]. The ‘sticky—spring’ model [31] assumes that, in the presence of calcium, titin’s PEVK (rich in proline (P), glutamic acid (E), valine (V), and lysine (K)) region can bind to actin. This leads to increased titin forces during and after active stretch. The resulting titin-induced half-sarcomere-based stresses have been homogenised and added to the continuum-mechanical stress tensor consisting of passive and active stress contributions. We used the proposed model to analyse muscle forces during and after active stretch starting on the plateau and the descending limb of the force—length relation by comparing the results obtained by the models with and without actin—titin interaction. In that contribution, we neglected the force—velocity relation.

In this study we demonstrate that actin—titin interactions [27–29] stabilise half—sarcomere lengths during fixed—length contractions and active stretches (neglecting inertia effects) on the descending limb of the force—length relation. Moreover, we demonstrate that the force—velocity relation does not stabilise half-sarcomeres in a similar way, as it has been discussed controversially in the past (cf. e. g. [33, 34]).

## Material and methods

**Fig 2:**
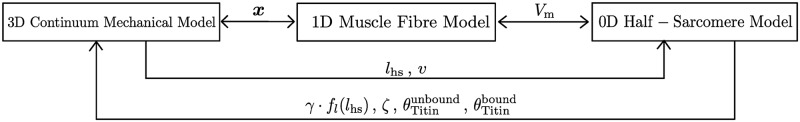
Overview of the multi-scale skeletal muscle model. Each box indicates a submodel. The coupling between the submodels is illustrated by the arrows in combination with the exchanged variables. (For further details see [19, 30, 32].)

In previous work [30], we included the biophysical ‘sticky—spring’ force enhancement model [31] into our continuum-mechanical skeletal muscle modelling framework [19, 32, 35]. Since this model is also the basis of the present work, we briefly review it here. Fig 2 provides a schematic overview of the presented multi-scale skeletal muscle model.

### The multi-scale muscle model

Muscles consist of muscle fibres arranged in parallel and these fibres are connected to each other laterally through the extracellular matrix, to which a significant portion of the fibre force can be transmitted [36, 37]. Thus, not only the arrangement of sarcomeres in series within a muscle fibre, but also their spatial arrangement within a muscle has to be considered to examine half-sarcomere—length inhomogeneities. Likewise, a large number of half-sarcomeres needs to be considered to obtain a statistically meaningful distribution. Consequently, a multi-scale continuum model is chosen in the present study.

**Fig 3:**
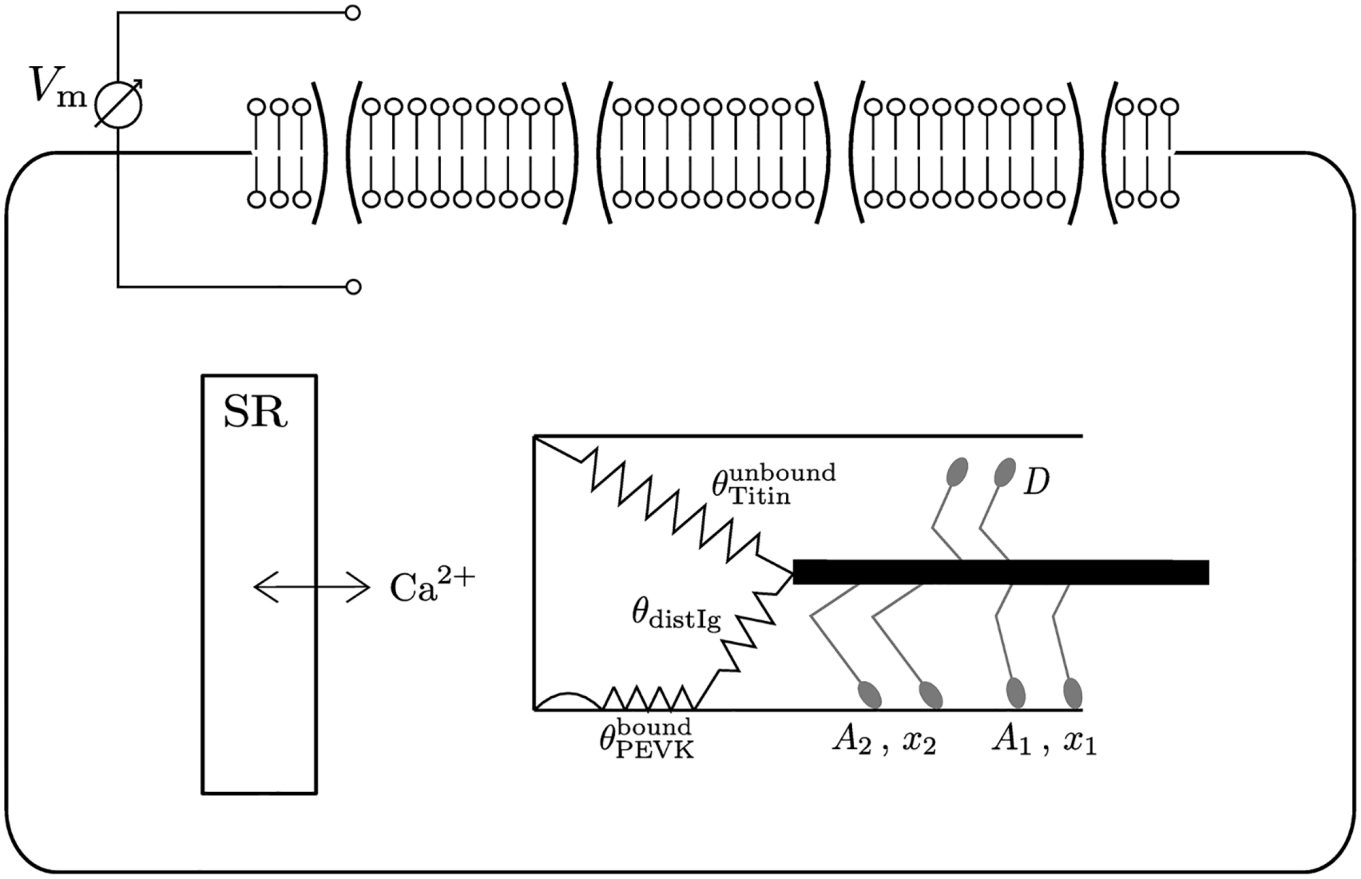
Schematic representation of the half-sarcomere model. The half-sarcomere model consists of a phenomenological description of the membrane voltage and the intracellular calcium concentration [41], that is coupled to a simplified Huxley-type model [42] and the ‘sticky—spring’ titin model [31].

The multi-scale skeletal muscle model [19, 32] embeds one-dimensional muscle fibre (finite element) meshes into the three-dimensional mesh of the muscle geometry. Note that the spatial locations of the computational half-sarcomeres, that make up the computational muscle fibres, is directly coupled to the actual (deformed) configuration of the three-dimensional continuum-mechanical model and hence enables force transmission along adjacent muscle fibres [37, 38]. The computational muscle fibres are used to compute the propagation of the action potential by solving the discrete monodomain equation [39]. The nonlinear reaction term of the monodomain equation is coupled to a model of the excitation—contraction coupling at the half-sarcomere level, describing the electro-physiology of the membrane, calcium release from the sarcoplasmic reticulum (SR), and cross-bridge cycling, cf. Fig 3. In previous works [19, 30, 32], we used a complex biophysical model [40] to simulate the excitation—contraction coupling on the cellular level. Since a detailed description of individual processes (e. g. the activity of single ion channels) is less important within the scope of this work, we replaced the biophysical model of the excitation—contraction coupling [40] with a phenomenological description of the membrane electro-physiology [41] coupled to a simplified Huxley-type model [42]. The coupling is realised by assuming that the slow variable of the phenomenological electro-physiological model [41] (a dimensionless representation of the conductance of a slow inward (repolarising) current [43]) behaves qualitatively similar to the calcium concentration in the myoplasm, which is required as input for the cross-bridge dynamics model [42]. Neglecting nearest-neighbour cooperative effects and distortion dependencies, the model predicts a linear force—velocity relation (see [42]). The linear approximation of the force—velocity relation is considered to be sufficient for the expected small range of contraction velocities due to the fact that only fixed-length contractions and quasi-static stretch experiments are considered in this work. The maximum shortening velocity of the muscle is assumed to be *v*_max_ = 15 *L*_0_/s [3, 44], where *L*_0_ denotes the fibre length of the resting muscle.

The resulting coupled model of the excitation—contraction coupling is solved at each discretisation point of the muscle fibre meshes [19]. The normalised half-sarcomere-based cross-bridge force, *γ*, and the binding probability of titin to bind to the actin filament, *ζ*, are computed from

$$\begin{array}{c} \gamma =\frac{A_{1}\;x_{1}+A_{2}\;x_{2}}{A_{2}^{\text{max}}\;x_{0}}\;\;\text{and}\;\;\zeta =\frac{A_{2}}{A_{2}^{\text{max}}}\;. \end{array}$$ (1)

Therein, *A*_1_ and *A*_2_ are the number of cross-bridges in the attached pre-power stroke and post-power stroke states, respectively, and *x*_1_ and *x*_2_ denote the corresponding average cross-bridge distortions. Further, $A_{2}^{\text{max}}$ is the number of cross-bridges in the attached post-power stroke state during an isometric tetanic contraction (stimulation frequency 100 Hz), and *x*_0_ denotes the cross-bridge distortion induced by the power-stroke under isometric conditions [42]. While the binding probability of titin, *ζ*, is assumed to be independent of the contraction velocity, the cross-bridge force is a function of the half-sarcomere velocity, i. e., $\gamma =\gamma ({\dot{l}}_{\text{hs}})$. In detail, *γ* = 0 indicates passive behaviour, and *γ* = 1 corresponds to an isometric contraction at a stimulation frequency of 100 Hz. The cross-bridge force is first scaled using the relation between active force and sarcomere length (*f*_*l*_) [1] and then homogenised ($T_{H}:\gamma f_{l}\to {\bar{\gamma f}}_{l}$) to be incorporated into the continuum-mechanical stress tensor, which is evaluated at the macroscale using the discrete representation of the three-dimensional muscle geometry. The homogenisation is also performed for the binding probability of titin ($T_{H}:\zeta \to \bar{\zeta }$).

Solving the balance of momentum at the macroscale, one obtains the contraction-induced deformation of the muscle geometry and the induced reaction forces. The local muscle deformation (represented by the fibre stretch, *λ*_f_) is interpolated ($T_{I}:\lambda_{f}l_{\text{hs}}^{\text{ref}}\to l_{\text{hs}}$) using the shape functions of the three-dimensional finite elements to the discretisation points of the muscle fibre meshes, where they are used to evaluate the microscopic force—sarcomere length relation *f*_l_ = *f*_l_(*l*_hs_) [1]. In the following, we refer to these interpolated lengths as half-sarcomere lengths, *l*_hs_. The resting half-sarcomere length in the undeformed, stress-free reference configuration (*λ*_f_ = 1) is assumed to be $l_{\text{hs}}^{\text{ref}}=1.0\;\mu \text{m}$ [11]. Further, the velocity is determined from the interpolated local muscle deformations, where an average over four time steps of the continuum-mechanical model (Δ*t*_mech_ = 0.1 ms) is computed to increase the numerical stability.

As in our previous work [30], we additionally solve a half-sarcomere-based force enhancement model [31] (the ‘sticky—spring’ model) at the discretisation points of the muscle fibre meshes. The ‘sticky—spring’ model assumes that in the presence of calcium titin’s PEVK region can bind to the thin filament, which reduces titin’s free molecular spring length to its distal Ig (immunoglobulin) region. The model distinguishes between titin filaments that are bound to actin, and titin filaments that are not bound to actin. The stress—stretch relations of both unbound and bound titin filaments is directly obtained from fitting experimental data [45, 46]. In the model, the stress in unbound titin filaments $\theta_{\text{titin}}^{\text{unbound}}$ results directly from this relation, i. e. $\theta_{\text{titin}}^{\text{unbound}}=\theta_{\text{titin}}^{\text{unbound}}\left(l_{\text{hs}}\right)$. The titin stresses induced by bound titin filaments is calculated by solving force equilibrium between titin’s distal Ig region and the actin—titin interconnection, i. e. *θ*_distIg_(*l*_hs_, *l*_hs[0]_) = *θ*_PEVK_(*l*_hs_, *l*_hs[0]_). (For further details, we refer to [31].) The ‘sticky—spring’ model assumes that the actin—titin interconnection behaves like a linear elastic spring, where the corresponding spring constant *k*_PEVK_ is a function of the half-sarcomere length at which titin was bound to actin *l*_hs[0]_. The relation *k*_PEVK_(*l*_hs[0]_) was determined from numerical simulations [31]. Consequently, the stress induced by bound titin filaments depends on the actual half-sarcomere length *l*_hs_ and the half-sarcomere length at which the titin filament was bound to the thin filament *l*_hs[0]_, i. e. $\theta_{\text{titin}}^{\text{bound}}=\theta_{\text{titin}}^{\text{bound}}\left(l_{\text{hs}},l_{\text{hs}}{}_{\left[0\right]}\right)$. Similar to the active stresses, the resulting titin stresses are homogenised ($T_{H}:\theta_{\text{titin}}\to {\bar{\theta }}_{\text{titin}}$) and added to the continuum-mechanical stress tensor. The resulting macroscopic first Piola-Kirchhoff stress tensor reads

$$\begin{array}{c} \begin{array}{cc} P\;=\; & -p\;\left(\det F\right)\;F^{T-1}\;+\;P_{\text{passive}}\left(F,M\right)\;+\\ & +\;P_{\text{active}}\left(\bar{\gamma },F,M\right)\;+\;P_{\text{titin}}\left(\bar{\zeta },{\bar{\theta }}_{\text{titin}}F,M\right)\;. \end{array} \end{array}$$ (2)

Therein, ***P***_active_ and ***P***_titin_ are the active and titin-induced stress contributions, where

$$\begin{array}{c} \begin{array}{cc} & P_{\text{active}}={\bar{\gamma f}}_{l}\;P^{\text{max}}I_{4}^{-1/2}FM\;\;\text{and}\\ & P_{\text{titin}}=\left[\bar{\zeta }{\bar{\theta }}_{\text{titin}}^{\text{bound}}+\left(1-\bar{\zeta }\right){\bar{\theta }}_{\text{titin}}^{\text{unbound}}\right]P^{\text{max}}I_{4}^{-1/2}FM\;, \end{array} \end{array}$$

(see [19, 30]). Further, ***P***_passive_ denotes the three-dimensional, transversely isotropic, hyperelastic passive stress tensor. Note that ***P***_passive_ is a smeared-out representation of the overall passive mechanical stiffness of the muscle including the extracellular connective tissue, passive contributions of the muscle fibres (e. g. cytoskeletal structures like nebulin or desmin) and matrix—fibre connectivity, but excludes the passive contribution of the titin filaments. This is realised by fitting the passive material parameters to experimental data from uniaxial compression tests, cf. [19]. Since within the scope of this publication we do not want to simulate a specific muscle we scaled the passive material properties (compared to [19, 32]) in order to obtain the property of a descending limb in the static total stress-stretch relation. Furthermore, (**·**)^*T*^ indicates a transposed tensor, ***F*** denotes the deformation gradient tensor, $M=a_{0}⊗a_{0}$ is a structural tensor, where ***a***_0_ denotes a referential unit vector pointing in the muscle fibre direction, *I*_4_ = ***a*** ⋅ ***a*** = ***Fa***_0_ ⋅ ***Fa***_0_ is the fourth (mixed) invariant of the right Cauchy-Green deformation tensor ***C*** = ***F***^*T*^ ***F*** and *P*^max^ is the maximum isometric tension. Finally, *p* is the hydrostatic pressure that enters the equation as a Lagrange multiplier due to the incompressibility constraint [47]. Note that the active and titin-induced stresses only act along the muscle fibre direction, while rather small [30] cross-fibre contributions are not considered. For material parameters of the titin model and the continuum-mechanical model, we refer to our previous publications (for the ‘sticky—spring’ model see [31], for the continuum-mechanical model see [30]). The entire model is implemented within the open-source software library OpenCMISS [48].

### Numerical simulations

To avoid influences of the geometry, a generic cubic muscle specimen with initial edge length *L*_0_ = 1cm is considered, in which the muscle fibres are aligned with one of the edges of the cube.

For the simulations, the muscle specimen is first passively stretched to a certain length *L*. Then, all embedded muscle fibres are simultaneously activated (tetanic stimulation, frequency 100 Hz) in their middle, while keeping the total length of the muscle specimen fixed. Note that individual segments of the simulated muscle are not constrained and can shorten against each other. Active forces are computed by subtracting the passive forces from the total forces obtained at a certain muscle length.

For the analysis, four models are compared: (i) model ${\text{M}}_{\text{ATI}}^{\text{Fv}}$ includes actin—titin interactions and the force—velocity relation, (ii) model M_ATI_ includes actin—titin interactions but omits the force—velocity relation, (iii) model M^Fv^ omits actin—titin interactions but includes the force—velocity relation, and (iv) model ${\text{M}}_{\text{x}}^{\text{x}}$ omits both actin—titin interactions and the force—velocity relation. In the models without actin—titin interaction, the titin-induced stresses do not vanish but describe the stresses induced by the titin filaments when they are not bound to actin, i. e., $P_{\text{titin}}={\bar{\theta }}_{\text{titin}}^{\text{unbound}}P^{\text{max}}I_{4}^{-1/2}FM$.

### Stability analysis

The following stability analysis considers the static equilibrium and thus neglects the force—velocity relation. In this case, *γ* and *ζ* coincide, i. e., *ζ* = *γ*, cf. Eq (1). Moreover, this analysis does not distinguish between macroscopic and microscopic quantities, i. e., $\bar{\left(\cdot \right)}=\left(\cdot \right)$ and $l_{\text{hs}}=\lambda_{f}=I_{4}^{1/2}$.

Within the theory of hyperelasticity, convexity guaranties the existence of a global minimiser and hence a unique solution. A sufficient condition for the existence of deformations minimizing a given hyperelastic potential *W*(***F***) = *W*(***F***, adj ***F***, det ***F***) is the polyconvexity condition:

$$\begin{array}{c} \begin{array}{cc} & \frac{\partial^{2}W}{\partial F⊗\partial F}\cdot \left(H⊗H\right)\;\geq \;0\;,\\ & \frac{\partial^{2}W}{\partial \mathrm{adj}F⊗\partial \mathrm{adj}F}\cdot \left(H⊗H\right)\;\geq \;0\;,\\ & \frac{\partial^{2}W}{\partial \det F^{2}}\;\geq \;0\;, \end{array} \end{array}$$ (3)

where ***H*** ≠ **0** denotes an arbitrary second-order tensor [49]. Regarding the stress tensor in Eq (2), the incompressible Mooney—Rivlin material and the anisotropic stress contribution making up for the first two terms on the right-hand side of Eq (2) are known to satisfy the polyconvexity condition (see Eq (3)) cf. [49, 50]. Further, due to the fact that the active and titin-induced stresses are not conservative, no strain energy can be defined for the last two terms on the right-hand side of Eq (2) [51, 52]. Rather than introducing pseudo-energies for these stress contributions, the definition of the first Piola—Kirchhoff stress tensor of the theory of hyperelasticity $P\;=\;\frac{\partial W}{\partial F}$ is used to eliminate the energy in Eq (3).

For the sake of readability, we introduce a short-hand notation for the active and titin-induced stress contributions:

$$\begin{array}{c} \hat{P}\;=\;\hat{S}\left(\gamma ,I_{4}\right)FM\;=\;P_{\text{active}}+P_{\text{titin}}\;, \end{array}$$ (4)

where $\hat{S}\left(\gamma ,I_{4}\right)$ denotes a scalar-valued function of the cross-bridge force *γ* and the fourth (mixed) invariant *I*_4_. Then, the active and titin-induced stress tensors satisfy the condition of Legendre—Hadamard ellipticity if

$$\begin{array}{c} \frac{\partial \hat{P}}{\partial F}\cdot \left(G⊗G\right)\;\geq \;0\;, \end{array}$$ (5)

where ***G*** ≠ **0** denotes an arbitrary second-order rank-one tensor, i. e., ***G*** = ***h*** ⊗ ***k*** ∀***h***, ***k***.

For a given cross-bridge force $\overset{*}{\gamma }$ and deformation $\overset{*}{I_{4}}$ the ellipticity condition can be written as [52]

$$\begin{array}{c} 2\;\overset{*}{I_{4}}\;\frac{d\hat{S}\left(\overset{*}{\gamma },\overset{*}{I_{4}}\right)}{dI_{4}}\;+\;\hat{S}\left(\overset{*}{\gamma },\overset{*}{I_{4}}\right)\;\geq \;0\;. \end{array}$$ (6)

To evaluate this condition, as a worst case scenario, we assume full activation ($\overset{*}{\gamma }=1$), such that

$$\begin{array}{c} \hat{S}\left(I_{4}\right)\;=\;I_{4}^{-1/2}P^{\text{max}}\left[f_{l}\left(I_{4}\right)+\theta_{\text{titin}}^{\text{bound}}\left(I_{4}\right)\right]\;. \end{array}$$ (7)

Inserting Eq (7) into Eq (6), and using $I_{4}^{-1/2}>0,\;P^{\text{max}}>0$, the sum of the active and titin-induced stress tensors satisfies the rank-one ellipticity condition if

$$\begin{array}{c} \frac{df_{l}\left(\overset{*}{I_{4}}\right)}{dI_{4}}+\frac{d\theta_{\text{titin}}^{\text{bound}}\left(\overset{*}{I_{4}}\right)}{dI_{4}}\;\geq \;0\;. \end{array}$$ (8)

Relation (8) states that the sum of the slopes of the active force—length relation and the titin-induced stress has to be positive for all muscle lengths. This condition is equivalent to the condition for a monotonically increasing scalar-valued function, due to the one-dimensional nature of the active and titin-induced stresses.

While the titin-induced stress $\theta_{\text{titin}}^{\text{bound}}$ is a monotonically increasing function [31], the active force—length relation [1] possesses a descending limb with negative stiffness. Keeping in mind that also the passive stress, which we omitted from this derivation, adds positive stiffness to the total behaviour, the result of the ellipticity condition can be interpreted as follows: the positive stiffness induced by the passive stresses and the titin-induced stresses have to compensate together for the negative stiffness on the descending limb of the force—length relation.

## Results

### The behaviour of the half-sarcomere model

**Fig 4:**
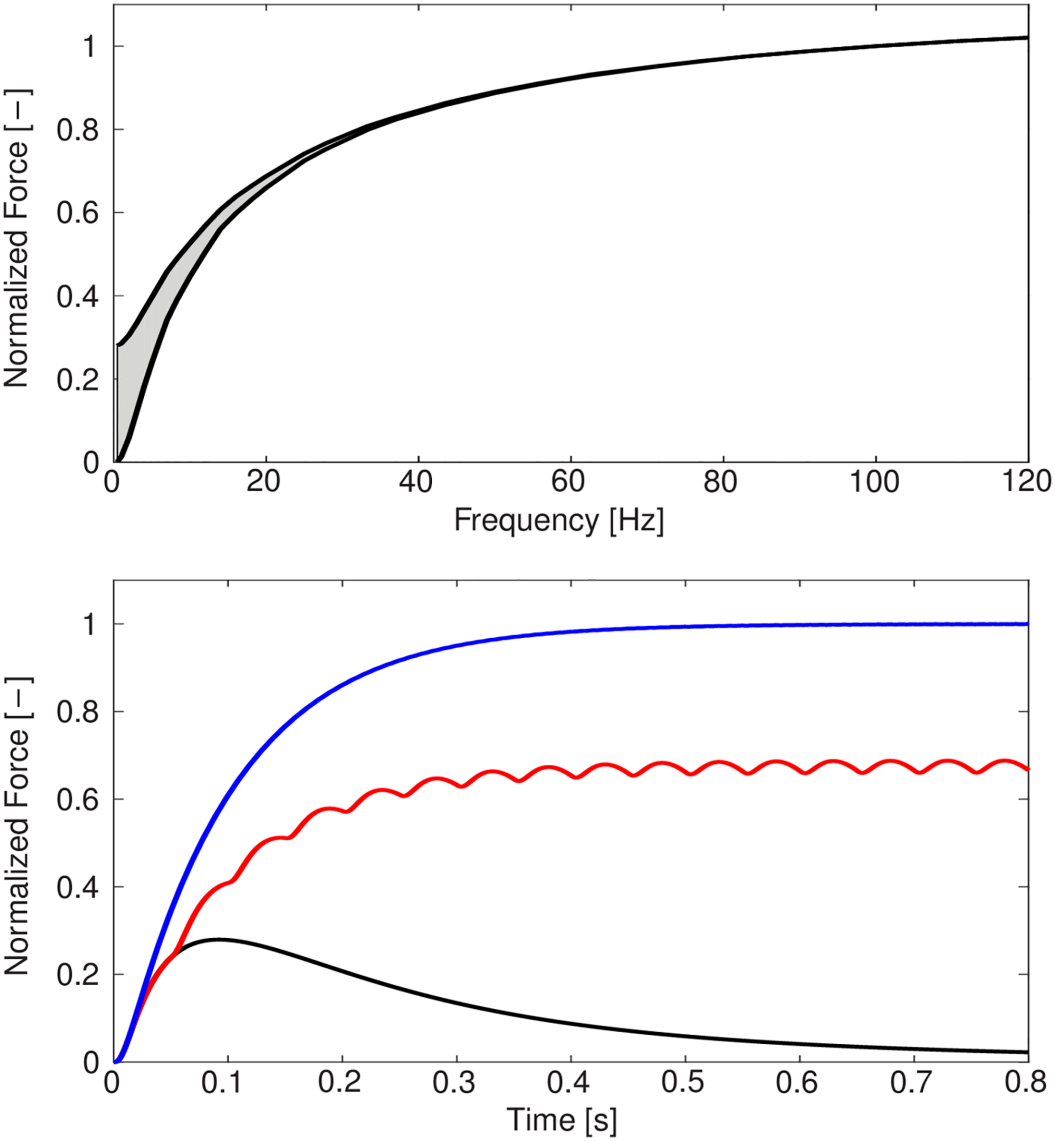
Top: Isometric force responses of the microscopic half-sarcomere model due to different stimulations. Single—twitch (black), submaximal contraction at 20 Hz (red), and tetanic contraction at 100 Hz (blue). Bottom: Force—frequency relation of the half-sarcomere model. Under conditions of not completely fused twitches, the range between the minimum and maximum values of the active force is shown.

First, we demonstrate that the newly introduced phenomenological half—sarcomere model of the excitation—contraction coupling is able to reproduce the expected physiological behaviour in response to stimulation. Fig 4 shows modelled isometric force responses due to different stimulation frequencies (top) and the model’s force—frequency relation (bottom). It can be observed that the model predicts a physiological twitch shape, twitch summation, and twitch-tetanus ratio.

**Fig 5:**
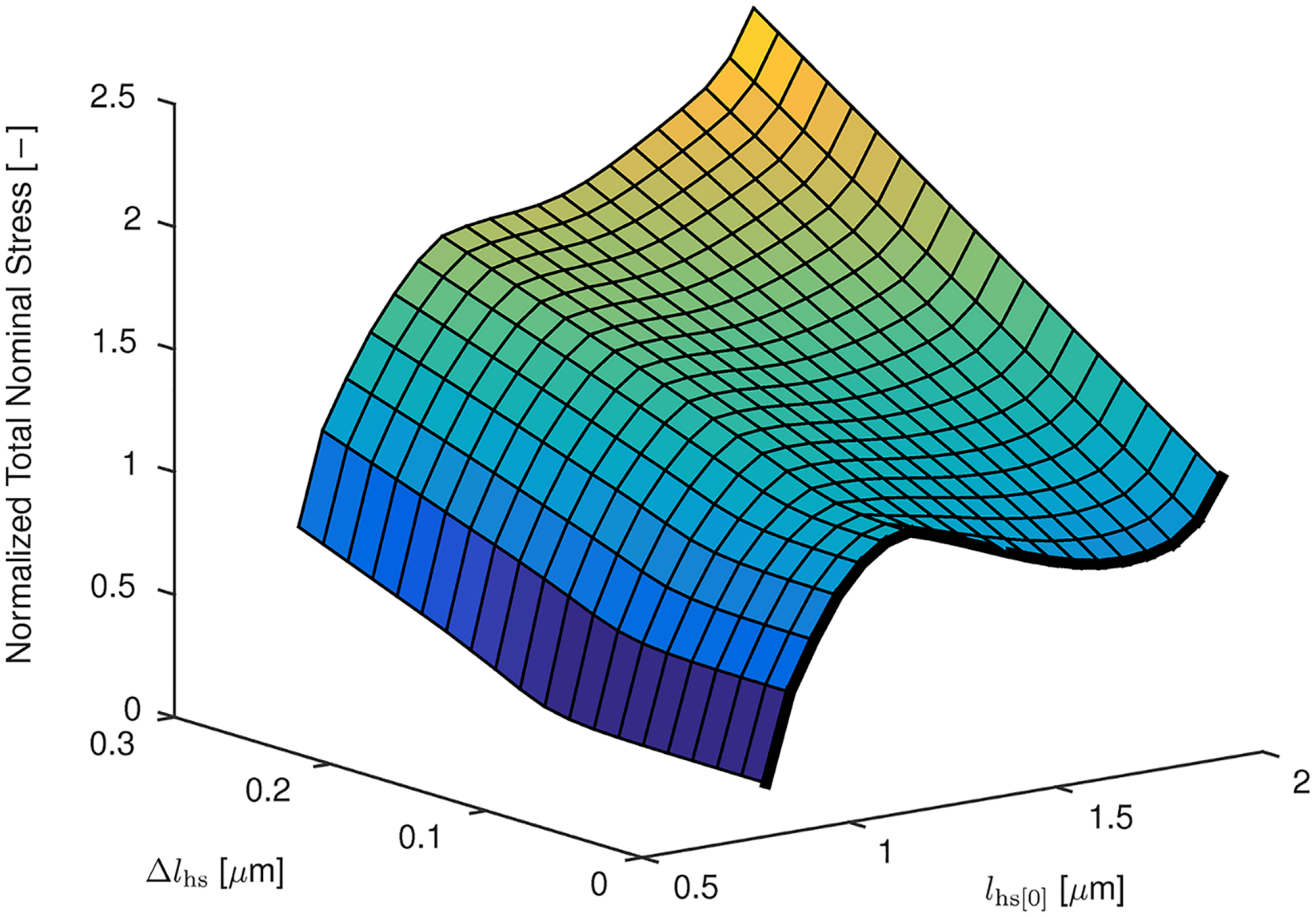
Total stress of the half-sarcomere model with actin—titin interaction versus the half-sarcomere length at which activation started, *l*_hs[0]_, and the active stretch increment, Δ*l*_hs_. The thick black curve at the right edge of the surface corresponds to the static total stress—stretch relation shown in Fig 8 (top).

Next, the quasi-static behaviour of the microscopic half-sarcomere model with actin—titin interaction is considered in isolation. To this end, isometric contractions have been carried out at different half-sarcomere lengths, *l*_hs[0]_. Moreover, for different initial half-sarcomere lengths, *l*_hs[0]_, i. e., the half-sarcomere lengths at which the activation started, quasi-static (i. e. the force—velocity relation was omitted) active stretch experiments have been performed for different active stretch increments, Δ*l*_hs_ = *l*_hs_ − *l*_hs[0]_. The results are summarised in a three-dimensional surface plot in Fig 5. While the relation between the total stress in a half-sarcomere and *l*_hs[0]_ (i. e., the total isometric force—length relation; thick black line in Fig 5) is not monotonically increasing, the total stress increases monotonically with the active stretch increment. In other words, the partial derivative of the total stress with respect to the active stretch increment is always positive, although the static total stress—stretch relation has a descending limb.

### Fixed-length contractions

**Fig 6:**
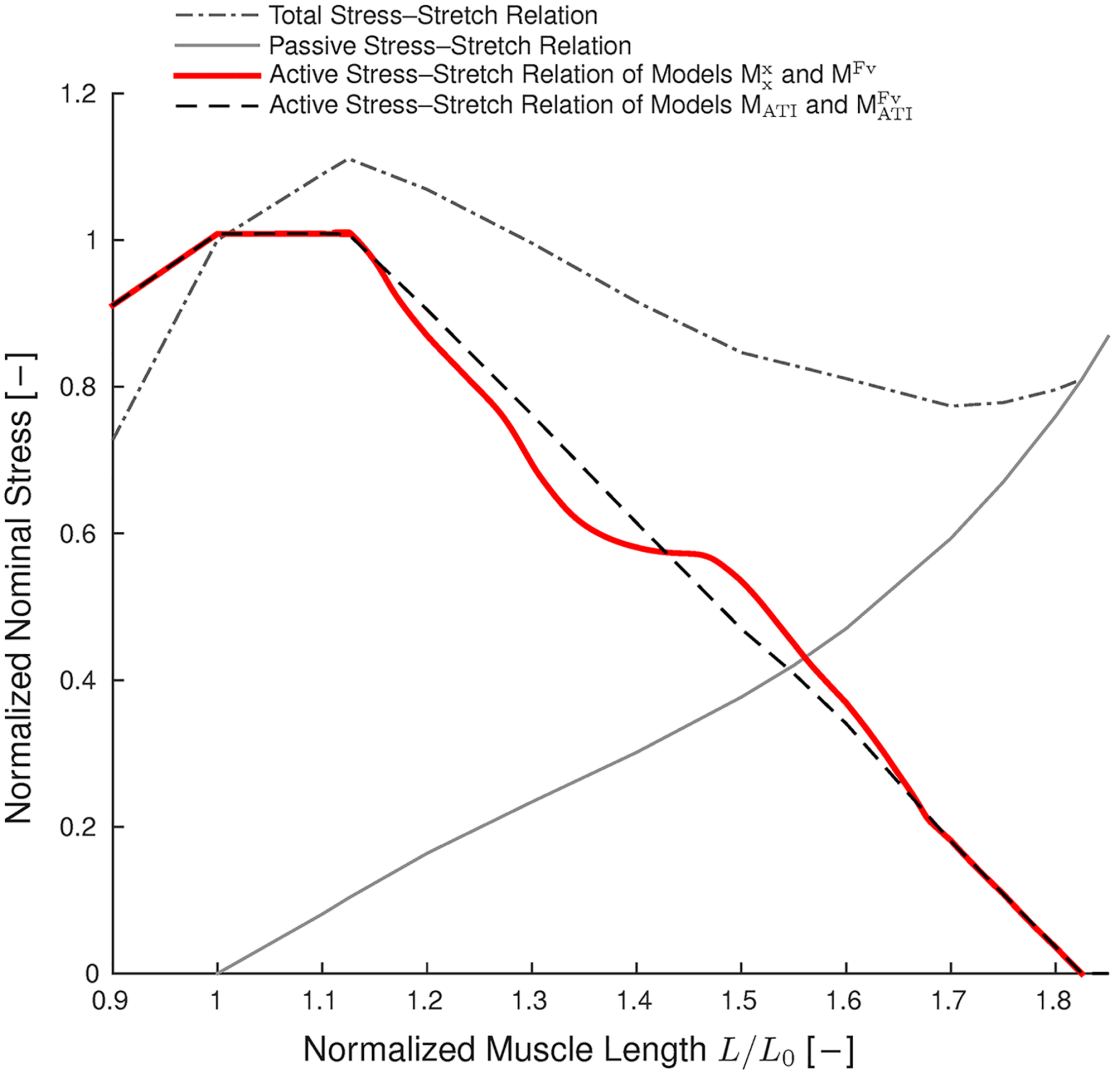
Isometric active force—length relations of the different models. Models ${\text{M}}_{\text{ATI}}^{\text{Fv}}$ and M_ATI_ reproduce the force—length relation as proposed in [1] that is included at the half-sarcomere level of all models. Models M^Fv^ and ${\text{M}}_{\text{x}}^{\text{x}}$ predict different forces on the descending limb. Under steady-state conditions (after 2.5 s) the results of models M^Fv^ and ${\text{M}}_{\text{x}}^{\text{x}}$, as well as those of models ${\text{M}}_{\text{ATI}}^{\text{Fv}}$ and M_ATI_, coincide.

Having established the behaviour of the microscopic half-sarcomere model, macroscopic whole-muscle simulations are investigated in the following. Fixed-length contractions at different muscle lengths have been carried out using the previously described four models, namely the ${\text{M}}_{\text{ATI}}^{\text{Fv}}$, M_ATI_, M^Fv^, and ${\text{M}}_{\text{x}}^{\text{x}}$ models. Fig 6 compares the resulting active force—length relations. Models ${\text{M}}_{\text{ATI}}^{\text{Fv}}$ and M_ATI_ reproduce the force—sarcomere length relation as proposed in [1] at the macroscopic whole muscle level, while models M^Fv^ and ${\text{M}}_{\text{x}}^{\text{x}}$ predict similar forces on the ascending limb and the plateau but deviating forces on the descending limb. In fact, there is a striking agreement between the stretch regions corresponding to the descending limb of the total stress—stretch relation and the occurrence of deviations from the expected linear force decrease in the models without actin—titin interaction (${\text{M}}_{\text{x}}^{\text{x}}$, M^Fv^). This analysis compares results after 2.5 seconds simulation time. At this time, transient effects caused by the force—velocity relation have disappeared and models M^Fv^ and ${\text{M}}_{\text{x}}^{\text{x}}$ predict similar results. At the very end of the descending limb of the total force—length relation and beyond (*L*/*L*_0_ > 1.6), the forces induced by bound titin filaments had to be relaxed by up to 25% to obtain convergence of models ${\text{M}}_{\text{ATI}}^{\text{Fv}}$ and M_ATI_. This can be explained by the large stiffness of the bound titin filaments at these lengths (cf. Fig. B1(A) of [31]) leading to numerical problems.

**Fig 7:**
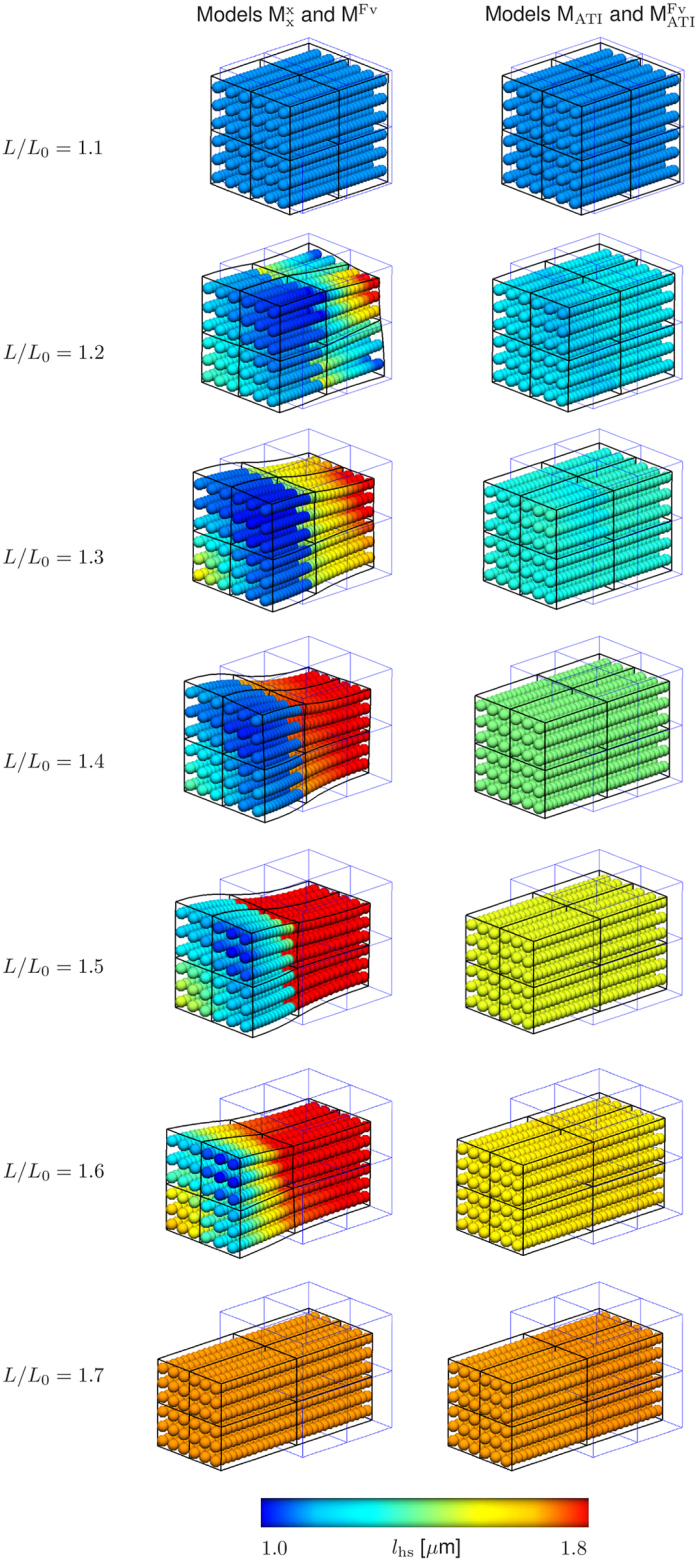
Distribution of half-sarcomere lengths, *l*_hs_, in the cubic muscle specimen for different muscle lengths ranging from *L*/*L*_0_ = 1.1 to 1.7 during fixed-length contractions of models ${\text{M}}_{\text{x}}^{\text{x}}$, M^Fv^, M_ATI_, and ${\text{M}}_{\text{ATI}}^{\text{Fv}}$. Results after 2.5 s are displayed (steady-state conditions), where the results of models ${\text{M}}_{\text{x}}^{\text{x}}$ and M^Fv^ (left), as well as those of M_ATI_ and ${\text{M}}_{\text{ATI}}^{\text{Fv}}$(right) coincide.

To further investigate the origin of the different behaviours of the models, Fig 7 shows the distribution of half-sarcomere lengths within the muscle specimen in fixed-length contractions at different muscle lengths after 2.5 seconds. While almost homogeneous half-sarcomere—length distributions are predicted by models ${\text{M}}_{\text{ATI}}^{\text{Fv}}$ and M_ATI_ for all muscle lengths, the models without actin—titin interaction predict homogeneous half-sarcomere—lengths only on the plateau and for very long muscle lengths, and strongly heterogeneous distributions on the descending limb of the total force—length relation.

**Fig 8:**
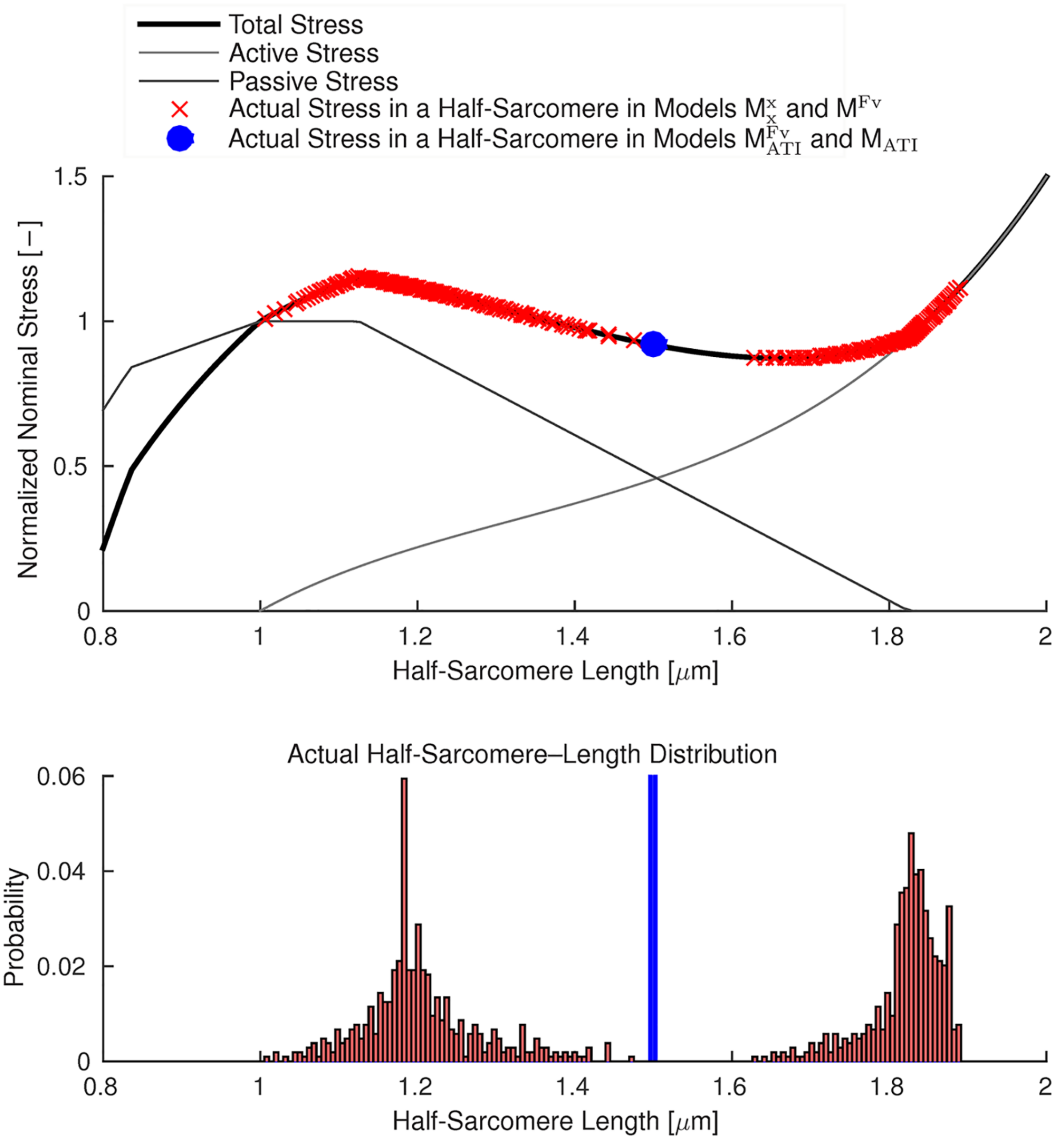
Top: Total, active, and passive force—length relations. Superimposed on the total stress curve are, for a muscle length of *L*/*L*_0_ = 1.5, the half-sarcomere lengths predicted by models ${\text{M}}_{\text{x}}^{\text{x}}$ and M^Fv^ (red crosses), and models M_ATI_ and ${\text{M}}_{\text{ATI}}^{\text{Fv}}$ (blue dot). The value predicted by models M_ATI_ and ${\text{M}}_{\text{ATI}}^{\text{Fv}}$ coincides with the theoretically predicted stress—stretch value. Bottom: Histogram of half-sarcomere lengths in models ${\text{M}}_{\text{x}}^{\text{x}}$ and M^Fv^ (red), and models M_ATI_ and ${\text{M}}_{\text{ATI}}^{\text{Fv}}$(blue, scaled for readability). Under conditions of steady state (after 2.5 s), the results of models ${\text{M}}_{\text{x}}^{\text{x}}$ and M^Fv^, as well as those of models M_ATI_ and ${\text{M}}_{\text{ATI}}^{\text{Fv}}$, coincide.

For the simulation with *L*/*L*_0_ = 1.5, Fig 8 (top) shows the stresses obtained with models ${\text{M}}_{\text{ATI}}^{\text{Fv}}$ and M_ATI_ (blue dot) and the distributions of half-sarcomere lengths obtained with models ${\text{M}}_{\text{x}}^{\text{x}}$ and M^Fv^ (red crosses) superimposed on the total force—length relation. Under steady-state conditions (after 2.5 s), the models without actin—titin interactions (M^Fv^, ${\text{M}}_{\text{x}}^{\text{x}}$) predict identical half-sarcomere length distributions that significantly deviate from the expected value of 1.5 *μ*m. In detail, models M^Fv^ and ${\text{M}}_{\text{x}}^{\text{x}}$ predict half-sarcomere lengths ranging from 1.0 *μ*m to 1.9 *μ*m, cf. Fig 8 (bottom).

Summarising the results shown in Figs 6, 7 and 8, a homogeneous half-sarcomere length distribution and active forces that are in accordance with the isometric force—length relation can only be obtained on the descending limb of the total force—length relation, when actin—titin interactions are included in the model. Further, it can be observed that the force—velocity relation cannot prevent the formation of half-sarcomere length inhomogeneities (models ${\text{M}}_{\text{ATI}}^{\text{Fv}}$ and M_ATI_, and likewise models M^Fv^ and ${\text{M}}_{\text{x}}^{\text{x}}$, predict similar results under steady-state conditions).

**Fig 9:**
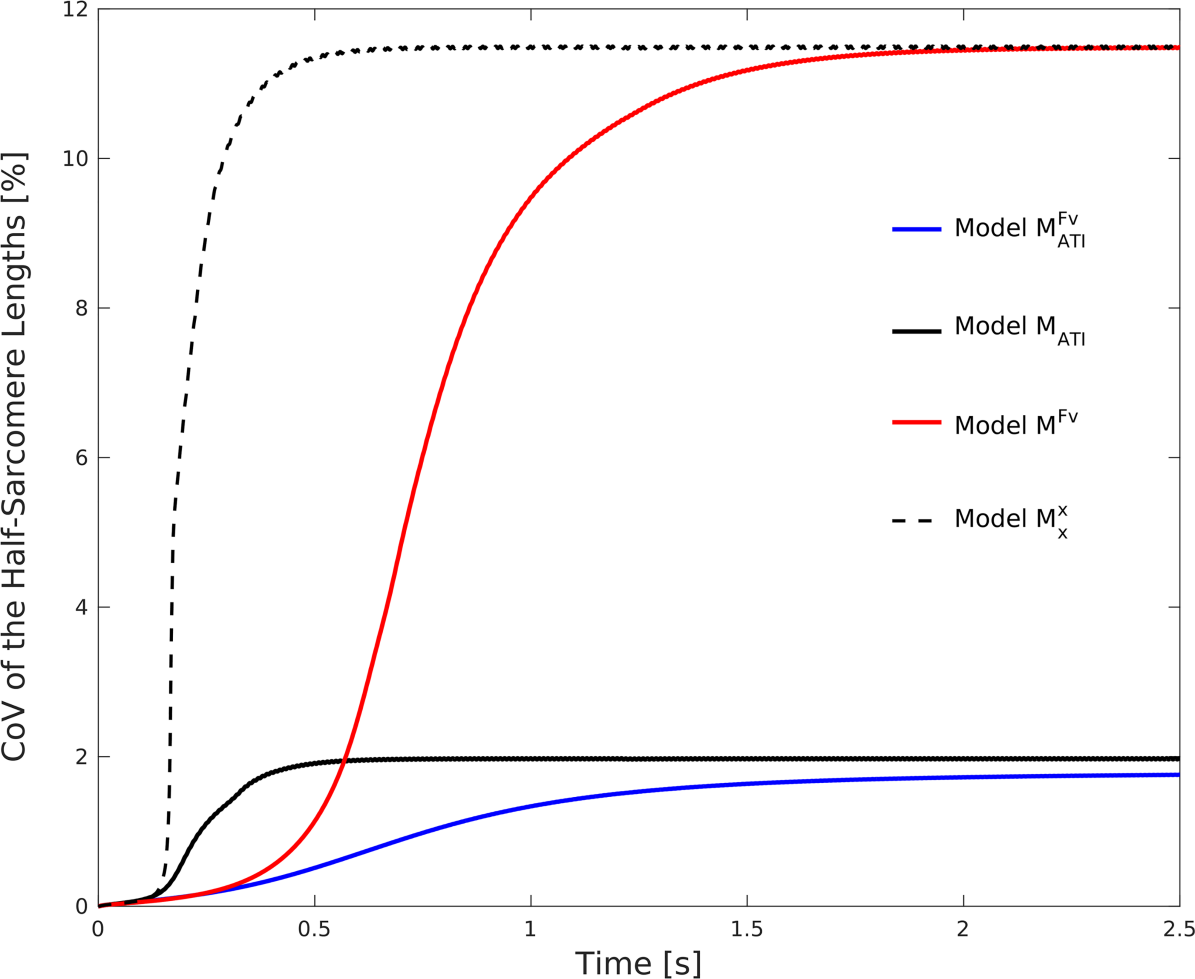
Coefficient of variation (CoV; standard deviation/mean*100%) of the half-sarcomere lengths during an isometric contraction at muscle length *L*/*L*_0_ = 1.2, for models ${\text{M}}_{\text{ATI}}^{\text{Fv}}$ (blue line), M^Fv^ (red line), M_ATI_ (black solid line) and ${\text{M}}_{\text{x}}^{\text{x}}$ (black dashed line). While actin—titin interactions can damp the magnitude of half-sarcomere—length heterogeneities, the force-velocity relation damps the their temporal evolution.

The previous results in this section considered steady-state results after 2.5 seconds, when transient effects caused by the force—velocity relation have disappeared. Fig 9 shows that the force—velocity relation delays the development of half-sarcomere—length heterogeneities. Note that the steepness of the force—length relation determines the rate of formation of half-sarcomere length heterogeneities, i. e. a steeper force—velocity relation decelerates the development of heterogeneities. For this analysis, the simulation with *L*/*L*_0_ = 1.2 is considered, in which inhomogeneities develop more slowly then at longer muscle lengths. While model ${\text{M}}_{\text{x}}^{\text{x}}$ predicts a high coefficient of variation (CoV; standard deviation/mean*100%) of the half-sarcomere lengths for the entire simulation time, the heterogeneities in model M^Fv^ appear only gradually but eventually reach the same high level. In contrast, model M_ATI_ predicts a constantly low coefficient of variation of the half-sarcomere lengths.

### Active stretch experiments

**Fig 10:**
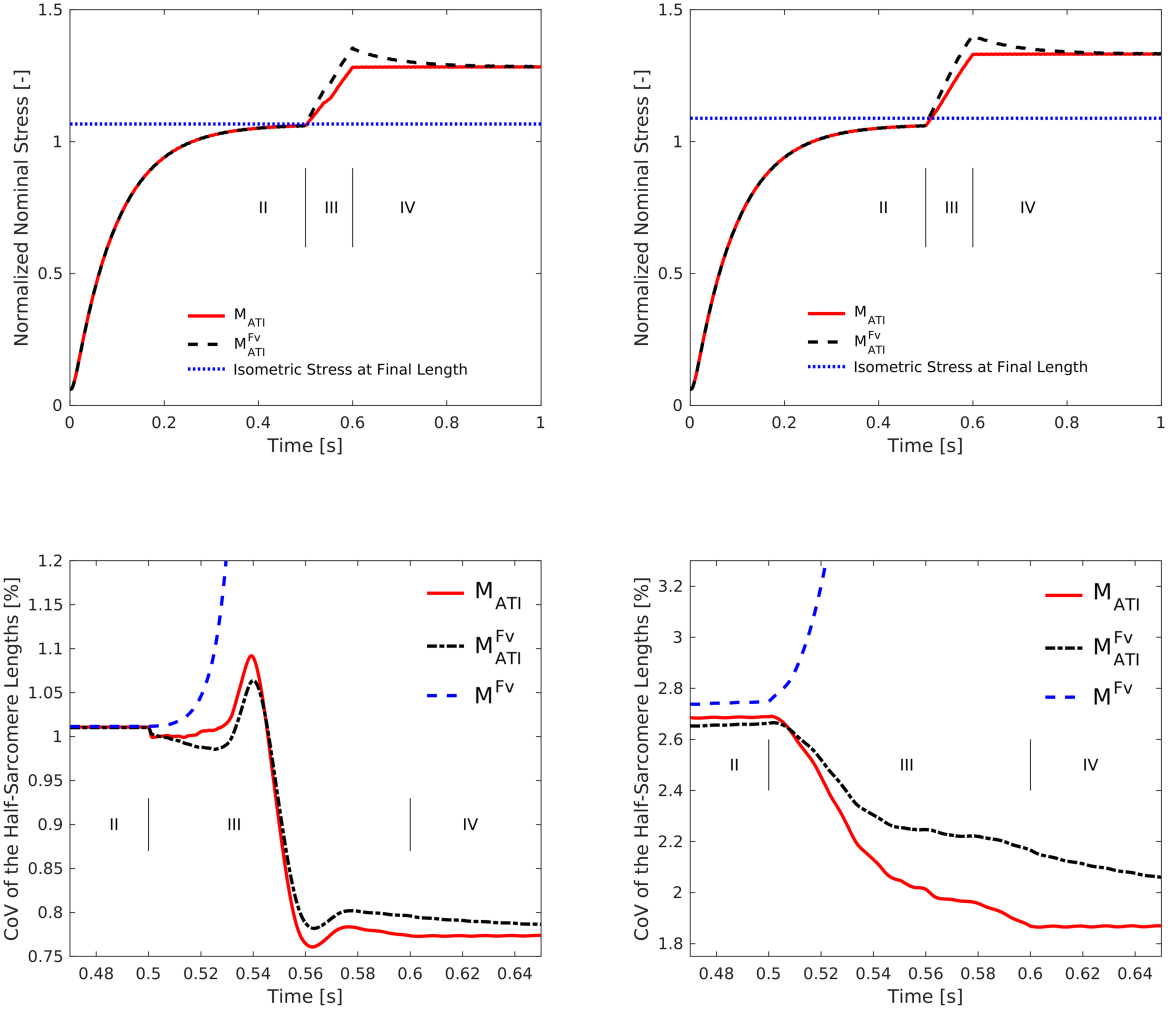
Active stretch experiments. Initial half-sarcomere length inhomogeneity is introduced via variation of passive (left column) and active (right column) material properties (see text). Top: Total stress evolution in models ${\text{M}}_{\text{ATI}}^{\text{Fv}}$ (black dashed line) and M_ATI_ (red line) following a protocol of passive stretch from *L*/*L*_0_ = 1.0 to 1.05 (phase *I*, i.e., computing the passive stretch as shown in Fig 6), full activation at fixed length (phase *II*), active stretch at a velocity of *v*/*v*_max_ = 13.3% from *L*/*L*_0_ = 1.05 to 1.25 (phase *III*), and subsequent fixed-length contraction (phase *IV*). Additionally, the stress resulting from a fixed-length contraction (model ${\text{M}}_{\text{ATI}}^{\text{Fv}}$) at the same final length *L*/*L*_0_ = 1.25 is shown (blue dotted line). The kink in the red line during the active stretch marks the transition from the plateau to the descending limb of the force—length relation. Note that we excluded stresses from models M^Fv^ and ${\text{M}}_{\text{x}}^{\text{x}}$ from these figures, since these models cannot reproduce the expected forces on the descending limb (previously shown, see e. g. Fig 6). Bottom: Coefficient of variation (CoV, standard deviation/mean*100%) of the half-sarcomere lengths corresponding to the above stretch experiments.

In order to test if the proposed model can predict the experimentally observed reduction in the half-sarcomere—length heterogeneity after active stretch [9], the previous model is slightly modified. To obtain an initially inhomogeneous distribution of the half-sarcomere lengths, we either introduced heterogeneous passive material properties or assumed that the maximum number of available cross-bridges per half-sarcomere shows random fluctuations. This was realised by scaling the corresponding material parameters (either the two isometric Mooney-Rivlin parameters and the two anisotropic parameters, or the maximum isometric force *P*_max_, for further details see [30]) in each finite element with a random value from a normal distribution with mean one and a standard deviation of 0.025. For both model variants, the initial passive stretch and the subsequent fixed-length contraction (phases *I* and *II*, Fig 10) yielded heterogeneous half-sarcomere lengths. For the model variant with variable passive properties the mean ± standard deviation of the half-sarcomere length distribution were 1.05 ± 0.01 *μ*m. For the model variant with variable active properties the mean ± standard deviation of the half-sarcomere length distribution were 1.05 ± 0.03 *μ*m. To verify that the linear approximation of the force—velocity relation is justified, the maximum shortening and lengthening half-sarcomere velocities occurring in these simulations have been analysed. They are in the range of |*v*| < 0.25 *v*_max_.

When modifying passive (Fig 10 left column) or active (Fig 10 right column) material properties, active stretch experiments show qualitatively similar results for total stresses (Fig 10 top row) and the evolution of the coefficient of variation (CoV) of the half-sarcomere lengths (Fig 10 bottom row) in models ${\text{M}}_{\text{ATI}}^{\text{Fv}}$, M_ATI_, and M^Fv^. The transient differences between models ${\text{M}}_{\text{ATI}}^{\text{Fv}}$ and M_ATI_ during and after the active stretch (phases *III* and *IV*) are caused by velocity-dependent effects. The differences (force enhancement) between the stresses of models ${\text{M}}_{\text{ATI}}^{\text{Fv}}$ and M_ATI_ compared to the stress of of a fixed length reference contraction (blue dotted line) at the same final length after 1 s (Fig 10 top) are evoked by actin—titin interactions (phase *IV*). The simulations of the model variant with distributed active properties show a higher magnitude of (residual) force-enhancement. This can be explained by the higher half-sarcomere length heterogeneity for the simulations with this model variant (cf Fig 10 bottom) and the resulting higher titin forces. When including actin—titin interactions, i. e. for models ${\text{M}}_{\text{ATI}}^{\text{Fv}}$ and M_ATI_, active stretch reduced significantly the CoV of the half-sarcomere lengths (cf. Fig 10 bottom). During phases *III* and *IV*, the CoV of the half-sarcomere lengths reduced by approximately 25% compared to its value before the active stretch. This behaviour deviates clearly from simulations with model M^Fv^, where the CoV of the half-sarcomere lengths monotonously increases first moderately and then rapidly to more than 20.

For both model variants, i. e. with either heterogeneous active or heterogeneous passive material properties, the CoV of the half-sarcomere lengths for models ${\text{M}}_{\text{ATI}}^{\text{Fv}}$ and M_ATI_ is not strictly decreasing and shows for all simulations parts with a positive time derivative (or at least equal zero). This behaviour is a consequence of the model assumption that the mechanical titin properties are more uniformly distributed than the passive and/or active material properties, i. e. in general the CoV of the half-sarcomere lengths decreases when the time derivative of the homogeneous distributed forces is greater than the time derivative of the heterogeneous distributed forces.

**Fig 11:**
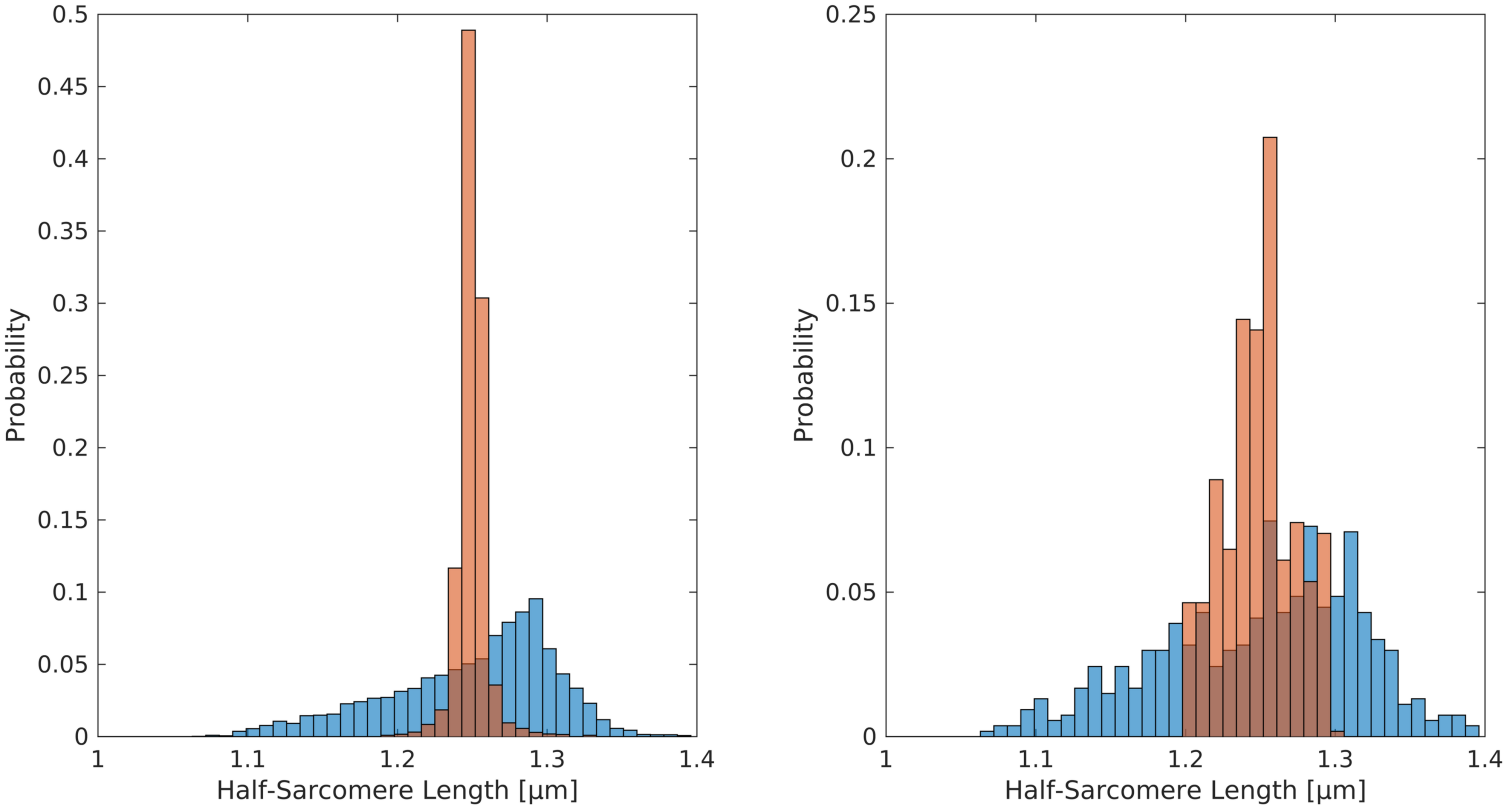
Histogram of half-sarcomere lengths in model ${\text{M}}_{\text{ATI}}^{\text{Fv}}$ with distributed material properties after an active stretch from *L*/*L*_0_ = 1.05 to 1.25 (red) and after a fixed-length contraction at the same final length *L*/*L*_0_ = 1.25 (blue). This figure compares steady-state results, i. e. transient effects of the force-velocity relation have disappeared. Left: Variable passive properties, right: Variable active properties.

Moreover, Fig 11 compares the final distribution of the half-sarcomere lengths resulting from the active stretch simulation (*L*/*L*_0_ = 1.05 to 1.25) of model ${\text{M}}_{\text{ATI}}^{\text{Fv}}$ with variable passive material parameters (cf. Fig 11 left) and variable active material parameters (cf. Fig 11 right) to the distribution of half-sarcomere lengths of a fixed-length contraction at *L*/*L*_0_ = 1.25 (blue). For both model variants the half-sarcomere lengths are less heterogeneously distributed after an active stretch than for a fixed length contraction at the same final length.

Thus, under the assumption that the material properties of titin are more homogeneously distributed than the passive and/or active material properties, the model can predict and explain, based on actin—titin interaction, the experimentally observed reduction in the half-sarcomere length heterogeneity after active stretch [9].

## Discussion

We analysed the effect of actin—titin interactions and the force—velocity relation on half-sarcomere lengths and muscle force during fixed-length muscle contractions and active stretches. Static convexity analysis revealed that the total stress—stretch relation including passive, active, and force enhancement-related contributions has to be monotonically increasing to obtain stability, and this prediction was confirmed by the numerical experiments. Thus, actin—titin interactions can be sufficient to obtain stable half-sarcomere operation, i. e. a nearly homogeneous half-sarcomere—length distribution, on the entire descending limb of the active force—length relation, and lead to active isometric forces in accordance with the descending limb of the classic force—length relation (Fig 6). In contrast, if actin—titin interaction is not included in the model, homogeneous half-sarcomere—lengths and active isometric forces in accordance with the descending limb of the force—length relation are generally not obtained, and unstable behaviour is observed during fixed-length contractions at lengths corresponding to the descending limb of the total force—length relation. The force—velocity relation can delay but not prevent the development of heterogeneities in the half-sarcomere length, and steady-state results coincide with those of the models without the force—velocity relation. The latter finding holds for both, models that include and models that do not include actin—titin interactions.

In the present paper, a simple cubic muscle without pennation was considered to minimize the potential influence of the muscle geometry [16, 53] on sarcomere lengths of adjacent muscle fibres and thus on our results. Thus, more homogeneous sarcomere lengths in adjacent muscle fibres can be expected from our model simulations (see Fig 7). In real muscle, complex geometries, which include, for example, elastic aponeuroses functioning as attachment areas for muscle fibres, various pennation angles, and different muscle compartments [54–56], might lead to different lengths of adjacent muscle fibres. These length differences will be much more pronounced during active contractions coupled with three-dimensional muscle deformation, changes in pennation angle, as well as storage and release of elastic energy due to the deformation of an aponeurosis in longitudinal and transversal direction. Recently, heterogeneous length changes along the fascicles of human medial gastrocnemius were reported during submaximal plantar flexion [57]. Due to the extracellular matrix (ECM), which provides elastic linkages between adjacent muscle fibres [58], local differences in the length of these adjacent fibres might result in development and transmission of local forces along the muscle fibre direction. Local forces in fibre direction can affect sarcomere length changes in general and thus can elevate or limit half-sarcomere length inhomogeneities. For example, in our model, the passive elastic properties led to coupling of half-sarcomere lengths, which can be observed in Fig 7 (left), where regions of distinct, relatively homogeneous half-sarcomere lengths developed during contractions at intermediate lengths (1.2–1.6 *μ*m). In summary, more complex muscle geometries might additionally influence sarcomere inhomogeneities on the descending limb of the force-length relation. To address this issue, implementation of more complex muscle architectures are required in future studies. However, modelling more complex geometries is straight forward within this finite element framework [35].

The distribution of half-sarcomeres during a fixed-length contraction at a length corresponding to the descending limb of the total force—length relation depends on the structure of the preparation and on the model. When considering a preparation consisting of only half-sarcomeres in series (e. g. a myofibril) and neglecting actin—titin interaction, half-sarcomeres would aggregate at two points on ascending albeit different limbs of the total force—length relation exhibiting the same total stress (Fig 1 top). The number of short and long half-sarcomeres would not be unique, and the distribution of the short and the long half-sarcomeres along the myofibril would be arbitrary. Since the continuum-mechanical model accounts additionally for transverse forces connecting the myofibrils and muscle fibres to each other in lateral directions, the half-sarcomere lengths obtained from simulations without actin—titin interaction are more diverse (Fig 7 left), and their frequency distribution shows two accumulation zones (Fig 8 bottom). Contrary to a myofibril, longer and shorter half-sarcomeres are grouped together in a muscle (Fig 7 left) due to forces of the passive matrix.

The calcium-induced binding of titin’s PEVK region to the actin filament reduces the molecular spring length of the titin filament, which significantly increases the passive stiffness of the half-sarcomere and yields a positive overall stiffness on its entire range of operation (including the entire descending limb of the active force—length relation, cf. Fig 5). The increased stability of the models including actin—titin interaction, which causes a homogeneous distribution of the half-sarcomere lengths in these models, can be attributed to the absence of a region with negative stiffness. Thus, the source of the theoretical instability, namely the imbalance between serially-arranged stronger (shorter) half-sarcomeres and weaker (longer) half-sarcomeres on the descending limb of the total force—length relation (cf. Fig 1), is absent in the models including actin—titin interactions (cf. Figs 6 and 7 right).

Further, actin—titin interaction leads to homogenisation of initially inhomogeneous half-sarcomere lengths during active stretch on the descending limb of the total force—length relationship in our models (Figs 10 and 11), an effect consistent with literature [9]. Assuming homogeneous titin properties across sarcomeres, bound titin is stiffer in initially longer half-sarcomeres [31]. (This result of the titin model is in accordance with recent muscle fibre experiments [59]). Hence, in subsequent stretches, titin forces increase disproportionately in half-sarcomeres, which are initially longer. This leads to disproportionate elongation of initially shorter half-sarcomeres and homogenisation of half-sarcomere lengths during active stretch compensating for initial inhomogeneities.

Within the scope of our study, the force—velocity relation delayed but did not prevent the development of half-sarcomere—length inhomogeneities on the descending limb of the total force—length relation (Fig 9). Thus, in general, the effect of actin—titin interaction on stability is different than that of the force—velocity relation. However, the force—velocity relation might stabilize the system for a short time (less than 0.5 s in our model, cf. Fig 9). The exact time, however, depends on the steepness of the force—velocity relation—steeper relations (lower values of *v*_max_) damp the system more effectively. We used a linear force—velocity relation, which is a reasonable approximation within the scope of the considered fixed-length contractions and quasi-static stretches.

The sarcomere—length non-uniformity theory [60–63] proposes that series—arranged (half-)sarcomeres can develop non-uniform lengths in muscle contractions due to the unstable behaviour on the descending limb of the force—length relation. Applying stretches beyond the plateau region of the force—length relation to a model of such half-sarcomeres in series resulted in non-uniform half-sarcomere lengths, and the subsequent isometric forces deviated from the theoretical sarcomere force—length relation [64], cf. Figs 6 and 7 (left). With the assumption of variations in the properties of half-sarcomeres within a myofibril (for example, strong and weak half-sarcomeres), this theory can also predict non-uniform half-sarcomere lengths and deviating forces in the range of the plateau region of the force—length relation.

However, the existence of sarcomere—length inhomogeneities in real muscle and predictions based on the sarcomere—length non-uniformity theory are a matter of debate [60, 65, 66]. A series of studies reported sarcomere—length inhomogeneities in myofibrils and muscle fibres after stretch [67–70] or even during fixed-length tetanic contractions [61]. In contrast, it has been reported that sarcomeres produce no instabilities on the descending limb [71]. Furthermore, it has been observed that, following stretch, half-sarcomere lengths were perfectly stable and sarcomere—length inhomogeneities were even reduced [9]. While the former results are in accordance with our simulations neglecting actin—titin interactions, the latter results are in accordance with our simulation results when including actin—titin interactions. Hence, actin—titin interaction might be particularly important for stable operation of muscles working on the descending limb of the force—length relation. Moreover, history- and activation-dependent actin—titin interactions can explain the phenomenon of force enhancement more completely than the sarcomere—length inhomogeneity theory [31] (especially forces exceeding the maximum isometric force by large amounts, e. g. [59, 72]).

The presumption that instabilities might exist on the descending limb results from generalising the force—length relationship (which is a static muscle property determined by a set of isometric experiments at different muscle lengths) to dynamic contractions. Many theoretical considerations and recent studies, for example, using Hill-type or continuum-mechanical muscle modelling, are based on this presumption by default. In view of the cited contradictory results with respect to the occurrence of sarcomere—length inhomogeneities in muscle contractions, this seems illicit at least for a subset of muscles. In fact, many experiments on whole muscle preparations [22, 73], isolated muscle fibres [9, 74, 75], and even single sarcomeres [76] reveal that the steady-state force following active stretch (when transient, velocity-dependent forces have vanished) on the plateau and the descending limb of the force—length relationship is higher than the force resulting from an isometric contraction at the same final length. This history-dependent behaviour, called (residual) force enhancement [22–24, 75], might be attributed to activation-dependent behaviour of the titin filament [27–29, 31, 66].

Taking all the available experimental findings into account, we conclude that, although there might be a descending limb in the isometric total force—length relation of a half-sarcomere/muscle, a half-sarcomere of a muscle exhibiting history-dependent effects never experiences negative stiffness (cf. the forces resulting from an active stretch increment in Fig 5), and thus instabilities and half-sarcomere heterogeneities are neither expected nor observed in such muscles [71]. Thus, the validity of mathematical models using hyperelastic stress—stretch relations that are based on the isometric force—length relationship describing these muscles has to be questioned.

### Conclusion

Our simulations show that actin—titin interactions can enable stable half-sarcomere operation on the descending limb of the active force—length relation during fixed-length muscle contractions and active stretches. Moreover, it has been demonstrated that the force—velocity relation can delay but not prevent the development of half-sarcomere length heterogeneities. Thus, actin—titin interactions and the force—velocity relation affect the stability of the system differently. It may be speculated that a key function of actin—titin interaction is to enable stable, predictable half-sarcomere operation avoiding large local stretches—that may for example damage the T-tubule system—for muscles working through the entire range of the force—length relation.

## References

[1] Gordon AM, Huxley AF, Julian FJ. The variation in isometric tension with sarcomere length in vertebrate muscle fibres. The Journal of Physiology. 1966;184:170–192. 10.1113/jphysiol.1966.sp007909 5921536 PMC1357553

[2] Rode C, Siebert T, Herzog W, Blickhan R. The effects of parallel and series elastic components on the active cat soleus force-length relationship. Journal of Mechanics in Medicine and Biology. 2009;9(01):105–122. 10.1142/S0219519409002870

[3] Siebert T, Leichsenring K, Rode C, Wick C, Stutzig N, Schubert H, et al Three-Dimensional Muscle Architecture and Comprehensive Dynamic Properties of Rabbit Gastrocnemius, Plantaris and Soleus: Input for Simulation Studies. PLOS ONE. 2015;10(6):e0130985 10.1371/journal.pone.0130985 26114955 PMC4482742

[4] Winters TM, Takahashi M, L LR, R WS. Whole muscle length-tension relationships are accurately modeled as scaled sarcomeres in rabbit hindlimb muscles. Journal of Biomechanics. 2011;44:109–115. 10.1016/j.jbiomech.2010.08.033 20889156 PMC3003754

[5] Huxley AF, Niedergerke R. Structural Changes in Muscle During Contraction: Interference Microscopy of Living Muscle Fibres. Nature. 1954;173:971–973. 13165697 10.1038/173971a0

[6] Huxley H, Hanson J. Changes in the cross-striations of muscle during contraction and stretch and their structural interpretation. Nature. 1954;173(4412):973–976. 10.1038/173973a0 13165698

[7] Hill AV. The mechanics of active muscle. Proceedings of the Royal Society of London B: Biological Sciences. 1953;141(902):104–117. 10.1098/rspb.1953.0027 13047276

[8] Edman KAP. The relation between sarcomere length and active tension in isolated semitendinosus fibres of the frog. The Journal of Physiology. 1966;183(2):407–417. 10.1113/jphysiol.1966.sp007873 5942818 PMC1357585

[9] Joumaa V, Leonard TR, Herzog W. Residual force enhancement in myofibrils and sarcomeres. Proceedings of the Royal Society of London B: Biological Sciences. 2008;275(1641):1411–1419. 10.1098/rspb.2008.0142 PMC2602709 18348966

[10] Burkholder TJ, Lieber RL. Sarcomere length operating range of vertebrate muscles during movement. The Journal of Experimental Biology. 2001;204:1529–1536. 11296141 10.1242/jeb.204.9.1529

[11] Edman KAP. The velocity of unloaded shortening and its relation to sarcomere length and isometric force on vertebrate muscle fibres. The Journal of Physiology. 1979;291:143–159. 10.1113/jphysiol.1979.sp012804 314510 PMC1280892

[12] ter Keurs HE, Iwazumi T, Pollack GH. The sarcomere length-tension relation in skeletal muscle. The Journal of General Physiology. 1978;72(4):565–592. 10.1085/jgp.72.4.565 309929 PMC2228549

[13] Prado LG, Makarenko I, Andresen C, Krüger M, Opitz CA, Linke WA. Isoform diversity of giant proteins in relation to passive and active contractile properties of rabbit skeletal muscles. The Journal of General Physiology. 2005;126(5):461–480. 10.1085/jgp.200509364 16230467 PMC2266601

[14] Wagner H, Siebert T, Ellerby DJ, Marsh RL, Blickhan R. ISOFIT: a model-based method to measure muscle—tendon properties simultaneously. Biomechanics and Modeling in Mechanobiology. 2005;4(1):10–19. 10.1007/s10237-005-0068-9 15895262

[15] Gareis H, Moshe S, Baratta R, Best R, D’Ambrosia R. The isometric length-force models of nine different skeletal muscles. Journal of Biomechanics. 1992;25(8):903–916. 10.1016/0021-9290(92)90230-X 1639834

[16] Blemker SS, Pinsky PM, Delp SL. A 3D model of muscle reveals the causes of nonuniform strains in the biceps brachii. Journal of Biomechanics. 2005;38(4):657–665. 10.1016/j.jbiomech.2004.04.009 15713285

[17] Röhrle O, Davidson JB, Pullan AJ. Bridging scales: a three-dimensional electromechanical finite element model of skeletal muscle. SIAM Journal on Scientific Computing. 2008;30(6):2882–2904. 10.1137/070691504

[18] Sharafi B, Blemker SS. A mathematical model of force transmission from intrafascicularly terminating muscle fibers. Journal of Biomechanics. 2011;44(11):2031–2039. 10.1016/j.jbiomech.2011.04.038 21676398 PMC3134549

[19] Heidlauf T, Röhrle O. Modeling the Chemoelectromechanical Behavior of Skeletal Muscle Using the Parallel Open-Source Software Library OpenCMISS. Computational and Mathematical Methods in Medicine. 2013;2013:1–14. 10.1155/2013/517287 PMC3855958 24348739

[20] Ehret AE, Böl M, Itskov M. A continuum constitutive model for the active behaviour of skeletal muscle. J Mech Phys Solids. 2011;59:625–636. 10.1016/j.jmps.2010.12.008

[21] Lemos RR, Epstein M, Herzog W, Wyvill B. A framework for structured modeling of skeletal muscle. Computer Methods in Biomechanics and Biomedical Engineering. 2004;7(6):305–317. 10.1080/10255840412331317398 15621651

[22] Abbott BC, Aubert XM. The force exerted by active striated muscle during and after change of length. The Journal of Physiology. 1952;117(1):77–86. 14946730 PMC1392571

[23] Rassier DE. The mechanisms of the residual force enhancement after stretch of skeletal muscle: non-uniformity in half-sarcomeres and stiffness of titin. Proceedings of the Royal Society of London B: Biological Sciences. 2012; p. rspb20120467. 10.1098/rspb.2012.0467 PMC3367795 22535786

[24] Minozzo FC, de Lira CAB. Muscle residual force enhancement: a brief review. Clinics. 2013;68(2):269–274. 10.6061/clinics/2013(02)R01 23525326 PMC3584266

[25] Rassier DE, MacIntosh BR, Herzog W. Length dependence of active force production in skeletal muscle. Journal of Applied Physiology. 1999;86(5):1445–1457. 10233103 10.1152/jappl.1999.86.5.1445

[26] Hill AV. The heat of shortening and the dynamic constants of muscle. Proceedings of the Royal Society of London B. 1938;126(843):136–195. 10.1098/rspb.1938.0050 18152150

[27] Bianco P, Nagy A, Kengyel A, Szatmári D, Mártonfalvi Z, Huber T, et al Interaction forces between F-actin and titin PEVK domain measured with optical tweezers. Biophysical Journal. 2007;93(6):2102–2109. 10.1529/biophysj.107.106153 17513381 PMC1959548

[28] Kellermayer MS, Granzier HL. Calcium-dependent inhibition of in vitro thin-filament motility by native titin. FEBS letters. 1996;380(3):281–286. 10.1016/0014-5793(96)00055-5 8601441

[29] Powers K, Schappacher-Tilp G, Jinha A, Leonard T, Nishikawa K, Herzog W. Titin force is enhanced in actively stretched skeletal muscle. The Journal of Experimental Biology. 2014;217(20):3629–3636. 10.1242/jeb.105361 25147246

[30] Heidlauf T, Klotz T, Rode C, Altan E, Bleiler C, Siebert T, et al A multi-scale continuum model of skeletal muscle mechanics predicting force enhancement based on actin—titin interaction. Biomechanics and Modeling in Mechanobiology. 2016;15(6):1423–1437. 10.1007/s10237-016-0772-7 26935301

[31] Rode C, Siebert T, Blickhan R. Titin-induced force enhancement and force depression: A ‘sticky-spring’ mechanism in muscle contractions? Journal of Theoretical Biology. 2009;259(2):350–360. 19306884 10.1016/j.jtbi.2009.03.015

[32] Heidlauf T, Röhrle O. A multiscale chemo-electro-mechanical skeletal muscle model to analyze muscle contraction and force generation for different muscle fiber arrangements. Frontiers in Physiology. 2014;5(498):1–14. 25566094 10.3389/fphys.2014.00498 PMC4274884

[33] Allinger TL, Epstein M, Herzog W. Stability of muscle fibers on the descending limb of the force-length relation. A theoretical consideration. Journal of Biomechanics. 1996;29(5):627–633. 10.1016/0021-9290(95)00087-9 8707789

[34] Zahalak GI. Can muscle fibers be stable on the descending limbs of their sarcomere length-tension relations? Journal of Biomechanics. 1997;30(11):1179–1182. 10.1016/S0021-9290(97)00079-1 9456388

[35] Röhrle O, Davidson JB, Pullan AJ. A physiologically based, multi-scale model of skeletal muscle structure and function. Frontiers in Physiology. 2012;3 10.3389/fphys.2012.00358 22993509 PMC3440711

[36] Monti RJ, Roy RR, Hodgson JA, Edgerton VR. Transmission of forces within mammalian skeletal muscles. Journal of Biomechanics. 1999;32(4):371–380. 10.1016/S0021-9290(98)00189-4 10213027

[37] Huijing PA. Muscle as a collagen fiber reinforced composite: a review of force transmission in muscle and whole limb. Journal of Biomechanics. 1999;32(4):329–345. 10.1016/S0021-9290(98)00186-9 10213024

[38] Pamuk U, Karakuzu A, Ozturk C, Acar B, Yucesoy CA. Combined magnetic resonance and diffusion tensor imaging analyses provide a powerful tool for in vivo assessment of deformation along human muscle fibers. Journal of the Mechanical Behavior of Biomedical Materials. 2016;63:207–219. 10.1016/j.jmbbm.2016.06.031 27429070

[39] Keener J, Sneyd J. Mathematical Physiology I: Cellular Physiology. vol. 1 2nd ed Antman SS, Marsden JE, Sirovich L, editors. Springer; 2009.

[40] Shorten PR, O’Callaghan P, Davidson JB, Soboleva TK. A mathematical model of fatigue in skeletal muscle force contraction. Journal of Muscle Research and Cell Motility. 2007;28(6):293–313. 10.1007/s10974-007-9125-6 18080210

[41] Aliev RR, Panfilov AV. A simple two-variable model of cardiac excitation. Chaos, Solitons & Fractals. 1996;7(3):293–301. 10.1016/0960-0779(95)00089-5

[42] Razumova MV, Bukatina AE, Campbell KB. Stiffness-distortion sarcomere model for muscle simulation. Journal of Applied Physiology. 1999;87(5):1861–1876. 10562631 10.1152/jappl.1999.87.5.1861

[43] Nash MP, Panfilov AV. Electromechanical model of excitable tissue to study reentrant cardiac arrhythmias. Progress in Biophysics & Molecular Biology. 2004;85(2):501–522. 10.1016/j.pbiomolbio.2004.01.016 15142759

[44] Luff AR. Dynamic properties of the inferior rectus, extensor digitorum longus, diaphragm and soleus muscles of the mouse. The Journal of Physiology. 1981;313:161–171. 10.1113/jphysiol.1981.sp013656 7277215 PMC1274442

[45] Linke A W, Ivemeyer M, Mundel P, Stockmeier R M, Kolmerer B. Nature of PEVK-titin elasticity in skeletal muscle. Proceedings of the National Academy of Sciences. 1998;95(14):8052–8057. 10.1073/pnas.95.14.8052 PMC20927 9653138

[46] Linke A W, Stockmeier R M, Ivemeyer M, Hosser H, Mundel P. Characterizing titin’s I-band Ig domain region as an entropic spring. Journal of cell science. 1998;111(11):1567–1574. 9580564 10.1242/jcs.111.11.1567

[47] Holzapfel GA. Nonlinear solid mechanics. John Wiley & Sons LTD, Chichester, West Sussex, England; 2000.

[48] Bradley CP, Bowery A, Britten R, Budelmann V, Camara O, Christie R, et al OpenCMISS: a multi-physics & multi-scale computational infrastructure for the VPH/Physiome project. Progress in Biophysics and Molecular Biology. 2011;107(1):32–47. 10.1016/j.pbiomolbio.2011.06.015 21762717

[49] Ball JM. Convexity conditions and existence theorems in nonlinear elasticity. Archive for rational mechanics and Analysis. 1976;63(4):337–403. 10.1007/BF00279992

[50] Markert B, Ehlers W, Karajan N. A general polyconvex strain-energy function for fiber-reinforced materials. Proc Appl Math Mech. 2005;5(1):245–246. 10.1002/pamm.200510099

[51] Rossi S, Ruiz-Baier R, Pavarino LF, Quarteroni A. Orthotropic active strain models for the numerical simulation of cardiac biomechanics. International Journal for Numerical Methods in Biomedical Engineering. 2012;28(6–7):761–788. 10.1002/cnm.2473 25364850

[52] Ambrosi D, Pezzuto S. Active stress vs. active strain in mechanobiology: constitutive issues. Journal of Elasticity. 2012;107(2):199–212. 10.1007/s10659-011-9351-4

[53] Zuurbier CJ, Huijing PA. Influence of muscle geometry on shortening speed of fibre, aponeurosis and muscle. Journal of Biomechanics. 1992;25(9):1017–1026. 10.1016/0021-9290(92)90037-2 1517262

[54] Böl M, Leichsenring K, Ernst M, Wick C, Blickhan R, Siebert T. Novel microstructural findings in M. plantaris and their impact during active and passive loading at the macro level. Journal of the Mechanical Behavior of Biomedical Materials. 2015;51:25–39. 10.1016/j.jmbbm.2015.06.026 26202470

[55] Siebert T, Tomalka A, Stutzig N, Leichsenring K, Böl M. Changes in three-dimensional muscle structure of rabbit gastrocnemius, flexor digitorum longus, and tibialis anterior during growth. Journal of the Mechanical Behavior of Biomedical Materials. 2017; 10.1016/j.jmbbm.2017.07.045 28778781

[56] Blemker SS, Delp SL. Three-Dimensional Representation of Complex Muscle Architectures and Geometries. Annals of Biomedical Engineering. 2005;33(5):661–673. 10.1007/s10439-005-1433-7 15981866

[57] Karakuzu A, Pamuk U, Ozturk C, Acar B, Yucesoy CA. Magnetic resonance and diffusion tensor imaging analyses indicate heterogeneous strains along human medial gastrocnemius fascicles caused by submaximal plantar-flexion activity. Journal of Biomechanics. 2017;57:69–78. 10.1016/j.jbiomech.2017.03.028 28433388

[58] Gillies AR, Lieber RL. Structure and function of the skeletal muscle extracellular matrix. Muscle & Nerve. 2011;44(3):318–331. 21949456 10.1002/mus.22094 PMC3177172

[59] Tomalka A, Rode C, Schumacher J, Siebert T. The active force—length relationship is invisible during extensive eccentric contractions in skinned skeletal muscle fibres. Proceedings of the Royal Society of London B: Biological Sciences. 2017;284 (1854). 10.1098/rspb.2016.2497 PMC5443931 28469023

[60] Edman KAP. Residual force enhancement after stretch in striated muscle. A consequence of increased myofilament overlap? The Journal of Physiology. 2012;590(6):1339–1345. 10.1113/jphysiol.2011.222729 22331422 PMC3382324

[61] Julian FJ, Morgan DL. Intersarcomere dynamics during fixed-end tetanic contractions of frog muscle fibres. The Journal of Physiology. 1979;293(1):365–378. 10.1113/jphysiol.1979.sp012894 315464 PMC1280718

[62] Morgan D. New insights into the behavior of muscle during active lengthening. Biophysical Journal. 1990;57(2):209–221. 10.1016/S0006-3495(90)82524-8 2317547 PMC1280663

[63] Morgan DL, Whitehead NP, Wise AK, Gregory JE, Proske U. Tension changes in the cat soleus muscle following slow stretch or shortening of the contracting muscle. The Journal of Physiology. 2000;522(3):503–513. 10.1111/j.1469-7793.2000.t01-2-00503.x 10713973 PMC2269772

[64] Campbell SG, Hatfield PC, Campbell KS. A mathematical model of muscle containing heterogeneous half-sarcomeres exhibits residual force enhancement. PLoS Comput Biol. 2011;7(9):e1002156 10.1371/journal.pcbi.1002156 21980268 PMC3182863

[65] Campbell SG, Campbell KB. Mechanisms of residual force enhancement in skeletal muscle: insights from experiments and mathematical models. Biophysical Reviews. 2011;3(4):199–207. 10.1007/s12551-011-0059-2 22180761 PMC3237401

[66] Herzog W, Powers K, Johnston K, DuVall M. A New Paradigm for Muscle Contraction. Frontiers in Physiology. 2015;6(174):1–11. 26113821 10.3389/fphys.2015.00174 PMC4461830

[67] Edman KAP, Tsuchiya T. Strain of passive elements during force enhancement by stretch in frog muscle fibres. The Journal of Physiology. 1996;490(1):191–205. 10.1113/jphysiol.1996.sp021135 8745287 PMC1158656

[68] Julian FJ, Morgan DL. The effect on tension of non-uniform distribution of length changes applied to frog muscle fibres. The Journal of Physiology. 1979;293(1):379–392. 10.1113/jphysiol.1979.sp012895 315465 PMC1280719

[69] Mutungi G, Ranatunga K. Sarcomere length changes during end-held (isometric) contractions in intact mammalian (rat) fast and slow muscle fibres. Journal of Muscle Research and Cell Motility. 2000;21(6):565–575. 10.1023/A:1026588408907 11206134

[70] Telley IA, Stehle R, Ranatunga KW, Pfitzer G, Stüssi E, Denoth J. Dynamic behaviour of half-sarcomeres during and after stretch in activated rabbit psoas myofibrils: sarcomere asymmetry but no ‘sarcomere popping’. The Journal of Physiology. 2006;573(1):173–185. 10.1113/jphysiol.2006.105809 16527855 PMC1618761

[71] Rassier DE, Herzog W, Pollack GH. Dynamics of individual sarcomeres during and after stretch in activated single myofibrils. Proceedings of the Royal Society of London B: Biological Sciences. 2003;270(1525):1735–1740. 10.1098/rspb.2003.2418 PMC1691424 12965002

[72] Leonard R T, Herzog W. Regulation of muscle force in the absence of actin-myosin-based cross-bridge interaction. American Journal of Physiology-Cell Physiology. 2010;299(1):C14–C20. 10.1152/ajpcell.00049.2010 20357181

[73] Herzog W, Leonard TR. Force enhancement following stretching of skeletal muscle: a new mechanism. Journal of Experimental Biology. 2002;205(9):1275–1283. 11948204 10.1242/jeb.205.9.1275

[74] Edman KAP, Elzinga G, Noble MI. Enhancement of mechanical performance by stretch during tetanic contractions of vertebrate skeletal muscle fibres. The Journal of Physiology. 1978;281(1):139–155. 10.1113/jphysiol.1978.sp012413 309001 PMC1282688

[75] Edman KAP, Elzinga G, Noble MI. Residual force enhancement after stretch of contracting frog single muscle fibers. The Journal of General Physiology. 1982;80(5):769–784. 10.1085/jgp.80.5.769 6983564 PMC2228643

[76] Leonard R T, DuVall M, Herzog W. Force enhancement following stretch in a single sarcomere. American Journal of Physiology-Cell Physiology. 2010;299(6):C1398–C1401. 10.1152/ajpcell.00222.2010 20844251

